# Inter-Organ Communication Networks in Systemic Physiology: Glucocorticoid Receptor α as a Central Integrator of Homeostasis

**DOI:** 10.3390/ijms27114702

**Published:** 2026-05-23

**Authors:** Gianfranco Umberto Meduri

**Affiliations:** Department of Medicine and Pharmaceutical Sciences, University of Tennessee Health Science Center, Memphis, TN 38163, USA; gmeduri@uthsc.edu

**Keywords:** glucocorticoid receptor alpha (GRα), inter-organ communication networks, systems-level homeostasis, immune–metabolic signaling, microbiome–host interactions, mechanotransduction, systems biology, endocrine regulation

## Abstract

The survival of complex multicellular organisms depends on continuous inter-organ communication networks that coordinate organism-wide responses across physiological conditions and stress states, including adaptation to environmental challenges, infection, and injury. Rather than operating as isolated units, organ systems are integrated through interconnected signaling networks that transmit biological information across tissues. Building on prior work examining individual physiological pathways, this review introduces a unified systems-level framework that integrates inter-organ communication into a coherent model of organism-wide regulation. This review proposes a systems-level framework in which homeostasis is maintained through eight principal communication systems: neural, endocrine, immune-inflammatory, vascular, lymphatic, metabolic, microbiome–gut, and mechanical-structural. Epithelial barriers function as dynamic signaling interfaces within multiple systems, while extracellular vesicles act as cross-system mediators of information transfer rather than as independent communication networks. These systems operate across distinct temporal scales to coordinate host defense, metabolic adaptation, vascular regulation, and tissue repair. The framework further introduces a temporal hierarchy of signaling dynamics that links communication systems to phase-specific responses during physiological stress. Within this integrated network, glucocorticoid receptor α (GRα) is proposed to function as a systems-level regulator of inter-organ communication, supported by converging mechanistic, experimental, and clinical evidence, with variability in the strength of evidence across domains. In contrast to prior reviews, which addressed GRα function within individual systems, this work conceptualizes GRα as a central rheostat coordinating cross-system signaling and temporal transitions in homeostatic correction. Evidence was identified through hypothesis-driven searches using the Consensus AI platform and verified through manual review of primary biomedical literature. GRα, a ligand-activated transcription factor expressed in most nucleated cells, enables hormonal stress signals to coordinate gene-expression programs across tissues, modulating neuroendocrine responses, endothelial function, inflammatory signaling, metabolic regulation, microbiome–host interactions, and tissue remodeling. Systemic responses to stress progress through three phases of homeostatic correction—Priming, Modulatory, and Restorative—within which GRα supports integrated organism-wide adaptation. This integrative framework provides a mechanistic basis for understanding the emergence and temporal evolution of biological responses in health and critical illness.

## 1. Introduction

This review examines the principal physiological communication systems through which organs exchange biological information to coordinate systemic adaptation and maintain homeostasis. Rather than treating organ systems as isolated units, it emphasizes interconnected networks—neural, endocrine, immune, vascular, lymphatic, metabolic, microbiome-gut, and mechanical—that collectively sustain integrated physiological regulation. Building on prior work examining individual components of glucocorticoid signaling, this manuscript introduces a unified systems-level framework that integrates these communication networks into a coherent model of organism-wide regulation. Particular attention is given to the role of glucocorticoid receptor α (GRα) as a systems-level regulator that integrates signaling across these networks under both basal and stress conditions. In this manuscript, the focus is on GRα, the functionally active isoform that mediates glucocorticoid receptor (GR) signaling. Although much of the literature refers more broadly to glucocorticoid receptor (GR) signaling without distinguishing receptor isoforms, GR signaling is interpreted here as predominantly mediated by GRα.

By integrating evidence from molecular biology, physiology, and critical care medicine, this review advances a network-based model of systemic regulation and examines how GRα-dependent signaling may coordinate transitions among the Priming, Modulatory, and Restorative phases of homeostatic correction [1]. In contrast to prior reviews, which addressed GRα function within specific biological domains, the present work synthesizes these elements into a comprehensive framework that explicitly links inter-organ communication systems with temporal phases of homeostatic correction. In this manuscript, “homeostasis” refers to the overall maintenance and restoration of internal physiological balance. However, many of the adaptive responses discussed—particularly during stress and critical illness—are more accurately characterized as allostatic processes, involving active, context-dependent adjustments of physiological systems to meet changing demands. In this context, allostasis denotes the dynamic regulatory mechanisms that maintain and re-establish homeostatic balance.

Within this model, organs function as interconnected signaling hubs rather than isolated units. GRα acts as a central molecular integrator, coordinating signals across communication networks to regulate organism-wide homeostasis under both basal conditions and physiological stress. This framework aligns with emerging systems biology approaches that conceptualize physiology as arising from interactions among interconnected biological networks rather than as a sum of organ-specific functions.

Recent transcriptomic and proteomic studies have identified distinct biological endotypes in sepsis and critical illness, highlighting substantial heterogeneity in host-response patterns [2,3]. While these approaches provide important descriptive classification, they do not fully explain how these biological states arise, interact, or transition over time [4]. These observations support the need for a systems-level framework that integrates inter-organ communication. In this context, the present model proposes that GRα may help coordinate these networks, providing a mechanistic basis for the emergence and temporal evolution of biological endotypes during homeostatic correction.

Evidence acquisition was conducted using a structured, hypothesis-driven query framework executed through the Consensus AI research platform, which aggregates peer-reviewed scientific literature primarily indexed in databases such as PubMed. Queries were prospectively designed and organized according to predefined inter-organ communication systems (vascular, neural, lymphatic, endocrine, immune, metabolic, microbiome, mechanical, and vesicle-mediated pathways), with an additional domain for cross-system integration.

These queries were developed to test whether GR signaling regulates or integrates key functional domains within each communication network. This approach enabled systematic interrogation of GR-mediated effects across biological systems and established a transparent, hypothesis-driven, reproducible strategy for evidence identification.

The structured query framework guiding AI-assisted evidence acquisition is summarized in Table 1. Retrieved studies were subsequently screened for relevance to predefined communication domains and for mechanistic specificity to GRα signaling. Priority was given to peer-reviewed studies providing direct or supportive evidence across experimental systems, including in vitro investigations, animal models, and human clinical or translational studies.

All studies identified through AI-assisted searches were manually verified against primary biomedical literature (PubMed-indexed sources) to ensure accuracy, relevance, and methodological validity, and key findings were cross-validated through independent review of primary sources. This combined approach enhances reproducibility while mitigating potential bias related to algorithmic retrieval, dynamic ranking, and non-versioned outputs.

This work represents a hypothesis-driven integrative synthesis rather than a formal systematic review; accordingly, limitations include potential selection bias and incomplete retrieval despite efforts to ensure comprehensive coverage.

### 1.1. Defining the Inter-Organ Communication Systems

The survival of complex multicellular organisms depends on the continuous exchange of biological information among organs, enabling coordinated responses to environmental challenges, infection, injury, and physiological stress. Through this exchange, spatially distributed organs function as an integrated physiological system.

This coordination is mediated by interconnected communication networks that link organs into a unified system, allowing signals generated in one tissue to influence distant sites and translate local perturbations into coordinated organism-wide adaptive responses.

These networks form organized inter-organ communication systems that regulate threat detection, energy allocation, perfusion, host defense, injury containment, and tissue repair. Rather than passive conduits, they transmit biologically encoded signals that determine how local events are integrated into systemic responses.

Inter-organ communication systems can therefore be defined as physiological networks that transmit information between tissues to preserve homeostasis under changing conditions. Although they differ in signaling speed, spatial reach, and duration, they operate simultaneously and interact continuously to maintain systemic regulation.

**Figure 1:**
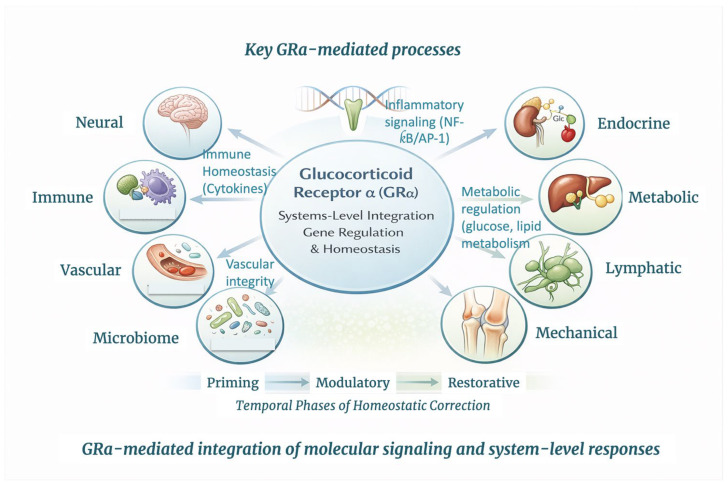
Systems-level integration of inter-organ communication networks by glucocorticoid receptor α (GRα). Legend: Schematic representation of the inter-organ communication network illustrating the eight principal communication systems—neural, endocrine, immune-inflammatory, vascular, lymphatic, metabolic, microbiome–gut, and mechanical–structural—and their dynamic interactions across multiple time scales. The figure highlights how signals are transmitted and integrated across tissues to coordinate systemic responses to stress, infection, and injury, and illustrates the proposed role of glucocorticoid receptor α (GRα) as a systems-level integrator of these interconnected signaling pathways. Extracellular vesicle signaling is represented as a cross-system mechanism that facilitates information transfer within and between communication domains. Figure 1 was created with assistance from ChatGPT (OpenAI, GPT 5) for conceptual organization and language refinement; all scientific content, interpretation, and final validation were performed by the author.

The principal inter-organ communication systems include neural, endocrine, immune, vascular, lymphatic, metabolic, microbiome–gut, and mechanical systems (Table 2). Extracellular vesicle signaling is not considered a distinct inter-organ communication system in this framework, but rather a cross-system mediator that facilitates information transfer among multiple domains, including immune-inflammatory, vascular, metabolic, and microbiome–gut systems. Figure 1 illustrates the systems-level organization of these inter-organ communication systems and their integration by GRα across the temporal phases of homeostatic correction. These eight inter-organ communication systems transmit diverse signals—including electrical impulses, neurotransmitters, hormones, cytokines, chemokines, circulating immune cells, metabolites, microbial products, interstitial fluid mediators, and mechanical forces—that enable coordinated information exchange across tissues.

Emerging concepts from evolutionary and developmental physiology suggest that homeostasis is a dynamic, diachronic process integrating development, physiology, and adaptation across the life cycle. Within this framework, cell–cell signaling networks form a continuous system that also contributes to evolutionary adaptation [29]. In this context, glucocorticoid signaling can be viewed as a conserved mechanism coordinating metabolic, immune, and vascular responses to stress, supporting the interpretation of GRα as a systems-level integrator linking molecular regulation to inter-organ communication.

Epithelial and endothelial barriers serve as dynamic structural and signaling interfaces embedded within multiple communication systems—particularly the microbiome, immune, and vascular systems—rather than constituting a separate communication system. These interfaces facilitate information transfer within and between communication domains.

Together, these inter-organ communication systems coordinate physiological responses across organs and are evolutionarily conserved as fundamental mechanisms of environmental sensing, energy regulation, host defense, and tissue repair. Although these systems operate continuously in health, critical illness provides a model of systemic failure, in which disruption of inter-organ communication affects inflammatory regulation, vascular integrity, endocrine coordination, metabolic allocation, and tissue repair.

At the center of this regulatory architecture, GRα acts as a ligand-activated, systems-level integrator, translating signals from multiple communication networks into coordinated transcriptional programs that govern organism-wide adaptation to stress and injury. This role reflects the near-ubiquitous expression of GRα across nucleated cells in virtually all organs and circulating immune cells (except enucleated erythrocytes) [1], allowing glucocorticoid signaling to regulate gene-expression programs across physiological systems.

The following sections examine these communication networks, their temporal organization, and the mechanisms by which GRα integrates them to coordinate systemic responses during physiological stress and critical illness.

### 1.2. Conceptual Framework of Inter-Organ Signaling Networks

Physiological regulation in multicellular organisms arises from coordinated communication among organs through interconnected signaling networks that transmit biological information across tissues and are functionally integrated by shared molecular regulatory systems. This framework shifts physiology from an organ-centered model to a network-based paradigm of systemic regulation.

Organ systems such as the respiratory, digestive, renal, and musculoskeletal systems function as physiological nodes—localized functional units that both generate and receive biological signals within the organism and serve as sites where systemic signals are produced, sensed, and integrated to regulate organ function. In this view, organs act as dynamic signaling hubs within distributed communication networks rather than isolated structures.

These hubs continuously exchange information through neural, endocrine, immune, vascular, lymphatic, metabolic, microbiome–gut, and mechanical pathways. Together, these communication systems form integrated networks that connect physiological nodes, enabling local events to generate coordinated organism-wide responses.

Recent advances in systems biology strongly support this network-based view. Large-scale transcriptomic studies, ligand–receptor interaction mapping, and inter-organ signaling analyses show that hormones, cytokines, metabolites, extracellular vesicles, and other circulating mediators form interconnected signaling networks linking distant tissues [30,31]. Extracellular vesicle–mediated transfer of proteins, lipids, and regulatory RNAs add an additional layer of inter-organ communication.

Through these distributed pathways, organs such as the liver, adipose tissue, skeletal muscle, gut, immune system, and brain function as coordinated signaling nodes that exchange biological information to maintain systemic homeostasis and adapt to physiological stress [32,33].

Inter-organ communication is not limited to circulating mediators. Bioelectrical signaling, calcium and ERK signaling waves, and mechanical cues also propagate information across tissues, coordinating processes such as growth, repair, and morphogenesis [34,35,36]. Mitochondria also function as cellular information processors, integrating metabolic and stress signals and transmitting them via reactive oxygen species, metabolites, and mitochondrial peptides [37]. At the organismal level, multi-tissue transcriptomic analyses reveal coordinated gene-expression programs linking distant organs and identify secreted mediators that participate in inter-organ communication [38,39].

Together, these findings demonstrate that organism-wide physiology emerges from densely interconnected, multi-scale signaling networks spanning endocrine, immune, metabolic, electrical, and mechanical systems.

In health, these communication networks are coordinated so that physiological adaptation remains proportionate, spatially restricted, temporally regulated, and reversible. In critical illness, however, this integration becomes disrupted: vascular signaling loses barrier precision, immune signaling becomes amplified or misdirected, endocrine timing becomes disturbed, metabolic allocation becomes inefficient, and repair pathways fail to synchronize across tissues. Consequently, signals that normally support adaptation instead drive systemic dysregulation.

A systems-based view of inter-organ communication provides a framework for identifying molecular regulators—such as glucocorticoid receptor α (GRα)—that integrate signaling across networks during stress, recovery, and restoration of homeostasis.

These inter-organ communication pathways can be organized into a coherent set of major communication systems that enable organs to exchange biological information across the body and coordinate organism-wide physiological regulation. For conceptual clarity, these systems can be categorized according to their primary signaling modality and their role in transmitting biological information between organs. Each system operates through distinct signaling mechanisms and temporal dynamics, yet all function together to support coordinated physiological regulation across tissues and organ systems. The principal inter-organ communication systems and their integration by GRα are summarized in Table 2 (Major Inter-Organ Communication Systems Coordinating Systemic Homeostasis and Their Integration by the Glucocorticoid Receptor α) [5,6,7,8,9,10,11,12,13,14,15,16,17,18,19,20,21,22,23,24,25,26,27,28]. These communication systems operate across a temporally organized continuum of signaling speeds, spatial reach, and functional roles, as summarized in Table 3.

Although each system is characterized by specific signaling modalities and kinetics, they function as an integrated network rather than as independent pathways. Through these interconnected networks—neural, endocrine, immune, vascular, lymphatic, metabolic, microbiome–gut, and mechanical—organs and tissues continuously exchange biological information, enabling coordinated systemic adaptation during physiological stress, illness, and recovery, and supporting the restoration and preservation of homeostasis. At the molecular level, GRα functions as a context-dependent regulator that coordinates these networks by integrating endocrine signals with local cellular responses across tissues.

Within this framework, GRα integrates convergent endocrine, immune, metabolic, and mechanical signals into coordinated transcriptional responses across tissues. This integrative function arises from its widespread cellular expression and its capacity to translate circulating hormonal signals into tissue-specific transcriptional programs shaped by local signaling environments. This systems-level role is consistent with GRα being a critical survival receptor required to integrate organism-wide adaptive responses that preserve homeostasis during physiological stress and critical illness [1].

This framework provides a network-based understanding of organism-wide physiology, in which multiple inter-organ communication networks converge on shared molecular regulators that coordinate systemic homeostasis. Viewed in this context, GRα functions not only as a mediator of endocrine glucocorticoid signaling but also as a molecular interface through which multiple communication systems are integrated to coordinate organism-wide adaptation to physiological stress. These systems operate across distinct temporal scales and functional roles, and their coordinated activity during physiological stress gives rise to dynamic, phase-specific patterns of systemic adaptation.

The temporal organization of these systems provides the mechanistic basis for the phase-specific progression of systemic responses during physiological stress. Across these transitions, GRα signaling coordinates communication across networks, ensuring that systemic responses stay coordinated, proportionate, and reversible. Within this framework, inter-organ communication unfolds across three phases of homeostatic correction [1].

During the Priming Phase, neural, vascular, immune, and metabolic signaling pathways rapidly mobilize energy resources, activate innate host defenses, and redistribute perfusion toward essential organs. As the response progresses to the Modulatory Phase, these networks shift to limit excessive inflammation, restore vascular barrier integrity, and optimize metabolic resource allocation to prevent collateral tissue injury. In the Restorative Phase, endocrine, metabolic, immune, and mechanical communication pathways promote tissue repair, extracellular matrix remodeling, and restoration of organ function. These phase-specific responses reflect coordinated, time-dependent activity across inter-organ communication systems operating over distinct temporal domains (Table 3).

Although these systems operate continuously to maintain homeostasis, their interactions become most evident during acute infection and inflammatory stress. In this setting, signals generated within one network rapidly propagate across others, producing coordinated organism-wide responses. Neural sensing pathways detect inflammatory signals and activate autonomic and neuroendocrine stress responses; immune signaling initiates cytokine-mediated communication across tissues; vascular and lymphatic systems regulate leukocyte trafficking and fluid distribution; endocrine pathways coordinate systemic metabolic and inflammatory regulation; and metabolic pathways mobilize energy substrates required to sustain immune and tissue responses. Microbiome-derived signals and mechanical forces generated by tissue injury further modulate these interactions.

Thus, during acute infection, inter-organ communication systems function as a tightly coupled, dynamically regulated physiological network [5,8,12,15,16,17,19,21,26,40,41,42,43,44,45,46]. Table 4 (Cross-System Coupling of Inter-Organ Communication Networks During Acute Infection) summarizes the principal systems, representative mediators, and key cross-system interactions linking local inflammatory events to coordinated systemic responses. 

The following sections examine each inter-organ communication system in detail, highlighting its signaling mechanisms, temporal organization, and GRα-mediated integration during phase-specific homeostatic correction.

### 1.3. Neural Communication System: Rapid Systemic Coordination

Among the inter-organ communication systems, neural signaling represents the fastest mechanism for coordinating organism-wide responses to environmental and internal stimuli.

#### 1.3.1. Hypothalamic–Pituitary–Adrenal Axis Integration

The neural communication network provides the fastest mechanism for inter-organ coordination, transmitting information through electrical impulses and neurotransmitter signaling within milliseconds. Through central and peripheral circuits, the nervous system integrates sensory inputs from the external environment and internal organs to regulate cardiovascular function, respiration, metabolic mobilization, thermoregulation, and behavioral responses to stress.

Neural communication is tightly coupled to endocrine signaling through the hypothalamic–pituitary–adrenal (HPA) axis, which links rapid neural sensing to systemic hormonal adaptation. Activation of hypothalamic corticotropin-releasing hormone (CRH) neurons stimulates pituitary adrenocorticotropic hormone (ACTH) release and subsequent glucocorticoid secretion from the adrenal cortex. Within this system, GRα signaling provides a critical negative-feedback mechanism that constrains HPA axis activation. GR activation by circulating glucocorticoids inhibits CRH neurons in the hypothalamic paraventricular nucleus through both rapid non-genomic and slower genomic mechanisms, thereby limiting HPA axis activity and maintaining stability of systemic stress responses [5,6,7]. Through this neuroendocrine interface, neural signals are rapidly translated into organism-wide hormonal responses, enabling coordinated regulation of immune, metabolic, and cardiovascular function during acute stress.

#### 1.3.2. Neural Regulation of Autonomic and Behavioral Stress Responses

GR signaling also integrates autonomic and behavioral stress responses across limbic and brainstem circuits. Glucocorticoid receptors expressed in forebrain regions—including the prefrontal cortex, hippocampus, and amygdala—mediate feedback regulation of psychogenic stress responses, whereas receptors in hypothalamic and brainstem nuclei regulate autonomic and endocrine components [47,48]. For example, in brainstem autonomic centers such as the nucleus of the solitary tract, GR signaling modulates autonomic outflow and neuroendocrine stress integration [40]. Through these region-specific actions, GRα integrates cognitive, emotional, and autonomic responses to support coordinated organism-wide adaptation to stress.

#### 1.3.3. Circadian Integration of Neural–Endocrine Signaling

Glucocorticoid signaling contributes to the temporal coordination of physiological responses through interactions with circadian regulatory systems. Circulating glucocorticoids display pronounced circadian and ultradian rhythms generated by the HPA axis, acting as systemic time-encoding signals that coordinate physiological activity across organs.

Through glucocorticoid receptor binding to response elements within core clock genes—including Period (PER) genes—glucocorticoids entrain peripheral circadian oscillators in tissues such as the liver, kidney, heart, and other metabolically active organs. These GR-dependent transcriptional effects synchronize local tissue clocks with central circadian signals from the suprachiasmatic nucleus [8,49,50].

Reciprocal regulation also occurs, as components of the molecular clock machinery, such as the CLOCK–BMAL1 complex, can modulate GR transcriptional activity, generating tissue-specific variations in glucocorticoid sensitivity across the circadian cycle [42,51].

In addition to central regulation of the HPA axis, glucocorticoids influence sympathoadrenal stress responses by regulating catecholamine synthesis in the adrenal medulla. Specifically, GR activation induces expression of phenylethanolamine N-methyltransferase (PNMT), the enzyme responsible for converting norepinephrine to epinephrine, thereby linking glucocorticoid signaling to the sympathoadrenal stress response [52,53,54].

Through these mechanisms, GRα coordinates neural, endocrine, and metabolic responses across temporal scales during acute stress. Together with the vascular system, neural signaling represents the fastest tier of inter-organ communication, initiating rapid organism-wide responses that are subsequently amplified and sustained by endocrine and immune signaling networks.

## 2. Endocrine–Hormonal Communication System 

### 2.1. Circadian Coordination of Peripheral Clocks

Glucocorticoids act as systemic circadian signals because their secretion follows a robust daily rhythm, and glucocorticoid receptors (GRs) are widely expressed in peripheral tissues, whereas the suprachiasmatic nucleus (SCN) is relatively insensitive to glucocorticoid signaling. Rhythmic glucocorticoid secretion, driven by coordinated SCN output and the adrenal peripheral clock, provides a daily endocrine timing signal that reaches virtually all tissues and organs. Association with immunophilins FKBP51 and FKBP52 determines receptor trafficking and signaling efficiency. FKBP52 facilitates dynein-mediated nuclear translocation and enhances GR transcriptional activity, whereas FKBP51 stabilizes cytosolic GR complexes, reduces ligand sensitivity, and delays nuclear import, thereby contributing to decreased glucocorticoid responsiveness. Through this coordinated chaperone system, GR signaling is dynamically regulated at the level of receptor activation, intracellular trafficking, and transcriptional output [55,56,57].

Following acquisition of a ligand-competent conformation, cytosolic GR translocates to the nucleus, where it binds glucocorticoid response elements (GREs) located in regulatory regions of core circadian clock genes. GREs have been identified in key circadian loci, including Per1, Per2, and Nfil3, allowing glucocorticoids to modulate the transcriptional feedback loops that generate circadian oscillations in peripheral tissues [8,9,10].

Experimental studies demonstrate that glucocorticoid administration can reset peripheral circadian oscillators in tissues such as the liver, kidney, heart, and immune cells without altering the master clock in the SCN. Through these mechanisms, glucocorticoids synchronize peripheral clocks with systemic circadian rhythms and integrate endocrine timing with metabolic and immune functions across organs [50,58,59,60]. These findings establish glucocorticoid signaling as a system-wide temporal regulator that coordinates circadian physiology across tissues.

Recent analyses further emphasize the central role of GRα in coordinating these systemic regulatory networks under health and critical illness conditions [1,61]. In this context, GRα functions as a temporal integrator, aligning circadian endocrine signals with tissue-specific transcriptional responses and synchronizing organism-wide physiological processes across organs [9,51].

### 2.2. Systemic Hormonal Coordination of Stress Responses

Beyond circadian regulation, glucocorticoids act as systemic endocrine signals that coordinate physiological responses to stress. By activating GRα across multiple organs, circulating glucocorticoids influence the mobilization of metabolic substrates, immune regulation, cardiovascular adaptation, and neuroendocrine feedback control. 

Because GRs are widely expressed, the glucocorticoid–GR axis enables rapid transmission of systemic regulatory signals across organs. This widespread receptor distribution allows endocrine glucocorticoid signaling to regulate gene-expression programs in nearly all tissues, supporting the role of GRα as a systems-level integrator linking endocrine signals with tissue-specific transcriptional responses during physiological stress [1].

## 3. Vascular Communication System: Endothelium as the Inter-Organ Signaling Bus

### 3.1. Systemic Vascular Network as a Communication Platform

Among the inter-organ communication systems, the vascular network occupies a unique position because it physically links all organs and tissues. Blood circulation not only transports oxygen, nutrients, hormones, cytokines, and metabolites, but also distributes immune cells, extracellular vesicles, and signaling molecules that convey physiological information across distant tissues. Thus, the vascular system functions as a system-wide communication platform through which local events in one organ can influence responses in distant organs.

### 3.2. Endothelial Cells as Distributed Signaling Interfaces

Within this network, the vascular endothelium serves as the principal sensing and regulatory interface. As previously described [62], endothelial cells line the entire circulatory system, maintain direct contact with circulating cells and plasma proteins, and regulate vascular tone, permeability, leukocyte trafficking, and microcirculatory flow through mediators such as nitric oxide, prostacyclin, endothelin, adhesion molecules, and glycocalyx-dependent signaling mechanisms.

They integrate hemodynamic forces with circulating endocrine, metabolic, and immune signals, enabling the vascular system to function as an active, distributed signaling interface that coordinates inter-organ communication rather than a passive transport pathway.

### 3.3. Endothelial Dysfunction and Breakdown of Vascular Communication

In critical illness, this communication interface becomes profoundly dysregulated [62]. Glycocalyx degradation, increased paracellular permeability, and enhanced leukocyte and platelet adhesion are hallmark features of endothelial dysfunction in conditions such as sepsis and acute respiratory distress syndrome (ARDS). This vasculocentric perspective emphasizes that endothelial injury not only accompanies organ failure but actively propagates maladaptive inter-organ cross-talk by disrupting perfusion, barrier integrity, inflammatory trafficking, and hemostatic balance.

GRα signaling helps maintain vascular communication by preserving endothelial barrier integrity, modulating inflammatory and coagulation pathways, and supporting microcirculatory function, thereby sustaining coordinated inter-organ signaling during physiological stress and critical illness [62].

### 3.4. GRα Regulation of Endothelial Barrier Integrity

GRα signaling plays a central role in preserving the integrity and regulatory capacity of this endothelial communication network. Activation of endothelial GRα strengthens junctional complexes by promoting the expression and stability of tight-junction and adherens-junction proteins, including occludin, claudin-5, ZO-1, and VE-cadherin, thereby limiting paracellular permeability and microvascular leak during inflammatory stress [11,12,13].

It also supports glycocalyx preservation and activates protective pathways such as sphingosine-1-phosphate kinase1 and angiopoietin-1/Tie2 signaling, further stabilizing endothelial barrier integrity and vascular homeostasis [12,13]. Through these mechanisms, GRα preserves endothelial barrier function and supports coordinated inter-organ communication during physiological stress and critical illness.

### 3.5. Regulation of Vascular Tone and Microcirculatory Perfusion

In parallel, GRα modulates vascular tone and microcirculatory perfusion through regulation of endothelial nitric oxide (NO) signaling pathways. Acute glucocorticoid signaling can activate endothelial nitric oxide synthase via rapid non-genomic GR–PI3K–Akt pathways, increasing nitric oxide availability and improving tissue perfusion [63,64]. Over longer time scales, GRα limits excessive nitric oxide production during systemic inflammation by restraining inducible nitric oxide synthase expression and inflammatory transcriptional activation [41,65]. Through these complementary actions, GRα dynamically regulates vascular tone and maintains microcirculatory perfusion and vascular homeostasis during physiological stress.

### 3.6. Suppression of Endothelial Inflammatory Activation

A third critical function of endothelial GRα signaling is the suppression of inflammatory activation. GRα directly interferes with NF-κB–dependent transcription through GR–RelA cross-talk (glucocorticoid receptor–NF-κB RelA/p65 interaction) and related transrepression mechanisms, reducing expression of adhesion molecules such as E-selectin, intercellular adhesion molecule 1 (ICAM-1), and vascular cell adhesion molecule 1 (VCAM-1) and limiting leukocyte adhesion to the vascular wall [13,66,67].

Experimental models demonstrate that loss of endothelial GR signaling results in prolonged NF-κB activation, excessive nitric oxide production, severe vascular dysfunction, and increased mortality during systemic inflammation [41]. Together, these findings establish GRα as an essential regulator of endothelial homeostasis that constrains maladaptive vascular inflammation and preserves inter-organ communication.

### 3.7. GRα as a Stabilizer of Vascular Communication

Through coordinated regulation of endothelial barrier integrity, perfusion control, and inflammatory trafficking, GRα stabilizes the endothelial communication interface, preserving the vascular system’s capacity to transmit physiological signals between organs during physiological stress and critical illness. In this context, GRα acts as a systems-level molecular integrator that sustains vascular communication and supports systemic homeostasis.

Beyond its endothelial actions, GR signaling also contributes directly to myocardial performance, supporting the hemodynamic component of vascular communication. Experimental studies demonstrate that cardiomyocyte GR activity is required for normal cardiac development, structural maturation, and maintenance of systolic function. Loss of cardiomyocyte GR signaling impairs ventricular contractility, promotes hypertrophy, reduces ejection fraction, and leads to premature heart failure in experimental models, underscoring its essential role in cardiac function. 

At the cellular level, GR regulates transcriptional programs controlling calcium handling, mitochondrial metabolism, and excitation–contraction coupling, including genes involved in oxidative metabolism and contractile function. Through these mechanisms, GRα supports the energetic and electrophysiological processes required for effective cardiac output and systemic perfusion. Balanced glucocorticoid and mineralocorticoid receptor signaling also appears critical for limiting pathological hypertrophic remodeling and fibrosis during cardiac stress. 

Together, these findings indicate that GRα supports vascular communication not only by stabilizing endothelial barrier integrity but also by preserving myocardial contractility and the hemodynamic forces required for effective tissue perfusion [68,69,70,71,72,73,74,75].

In addition, GR signaling enhances cardiovascular responsiveness to catecholamines by upregulating adrenergic receptor expression and maintaining vascular smooth muscle sensitivity to vasoconstrictor stimuli, thereby supporting systemic vascular tone and circulatory stability during physiological stress and critical illness [41,75,76,77].

Collectively, endothelial signaling, myocardial contractility, and vascular smooth muscle responsiveness function as coordinated components of the vascular communication network, through which GRα preserves systemic coordination during stress.

Vascular regulation, however, represents one component of the broader inter-organ communication framework (Table 2), within which GRα integrates signaling across immune–inflammatory, endocrine, metabolic, and epithelial barrier systems to balance host defense, energy allocation, and tissue repair.

Through these distributed actions, GRα functions not simply as an anti-inflammatory regulator but as a systems-level integrator that stabilizes vascular communication, preserves systemic perfusion, and coordinates organism-wide adaptive responses during physiological stress and critical illness.

## 4. Lymphatic Communication System 

### 4.1. Lymphatic Network as an Inter-Organ Communication Pathway

The lymphatic system provides a specialized pathway for the transport of immune cells, inflammatory signals, lipids, and microbial antigens from peripheral tissues to lymph nodes and the systemic circulation. Through this network, it integrates local tissue signals with systemic immune and metabolic responses, contributing to host defense, fluid homeostasis, and inflammatory regulation. In this context, lymphatic vessels function as a distributed inter-organ communication conduit linking peripheral tissues with central immune compartments.

### 4.2. GRα Regulation of Lymphangiogenic Signaling

GRα signaling regulates lymphatic communication by modulating lymphangiogenic pathways and lymphatic endothelial cell function. Experimental studies demonstrate that glucocorticoids suppress the expression of vascular endothelial growth factor-C (VEGF-C), a principal driver of lymphatic vessel growth, through GRα–dependent transcriptional repression mechanisms.

In tumor and inflammatory models, glucocorticoid treatment reduces VEGF-C expression and lymphatic vessel formation while largely preserving expression of the primary lymphatic receptor VEGFR-3, indicating that regulation occurs predominantly at the ligand level rather than through direct receptor modulation [14]. This ligand-centric mechanism of control constrains pathological lymphatic expansion while maintaining baseline lymphatic responsiveness.

### 4.3. Lymphatic Communication During Inflammation and Tissue Remodeling

Additional experimental and imaging studies demonstrate that lymphangiogenesis is activated during inflammatory tissue remodeling and wound healing, processes largely driven by VEGF-C–dependent signaling pathways [15,16]. Because glucocorticoids suppress multiple inflammatory pathways, GRα activation limits inflammation-induced VEGF-C expression, thereby restraining lymphatic expansion during tissue remodeling.

### 4.4. Role of the Lymphatic System in Systemic Immune Communication

Through these mechanisms, glucocorticoid signaling coordinates lymphatic-mediated inter-organ communication by controlling lymphatic vessel growth, inflammatory mediator transport, and immune cell trafficking. Within the broader inter-organ communication framework, the lymphatic network functions as a critical conduit linking local tissue inflammation with systemic immune and metabolic regulation. While this regulation limits excessive inflammation and lymphatic remodeling, it may also reduce antigen transport and immune surveillance, highlighting a context-dependent trade-off in GRα-mediated regulation of lymphatic communication.

## 5. Immune–Inflammatory Communication System: Coordinating Host Defense

### 5.1. Immune Signaling as an Inter-Organ Communication Network

The immune–inflammatory network constitutes a central inter-organ communication system that coordinates host defense and tissue responses to infection and injury. Through the release of cytokines, chemokines, alarmins, and other inflammatory mediators, immune cells transmit signals between tissues that regulate leukocyte recruitment, pathogen clearance, and activation of innate and adaptive immunity. These signaling pathways allow localized tissue injury or infection to generate coordinated systemic responses across vascular, neural, endocrine, and metabolic networks. Within this framework, immune signaling functions as a dynamic and adaptive communication axis that integrates local inflammatory cues with organism-wide physiological adaptation.

### 5.2. GRα Regulation of Inflammatory Transcriptional Networks

GRα signaling plays a central role in regulating this immune communication network by transcriptionally controlling inflammatory gene expression. Activated GRα suppresses inflammatory signaling through direct interactions with key transcription factors, including nuclear factor κB (NF-κB) and activator protein-1 (AP-1). Through this cross-talk, GRα inhibits transcription of inflammatory mediators—including cytokines, chemokines, adhesion molecules, and matrix metalloproteinases—thereby limiting amplification and promoting coordinated resolution of inflammation across interconnected tissues and organ systems [17,18,19].

Glucocorticoid receptor β (GRβ), an alternatively spliced isoform that does not bind glucocorticoids, provides an additional regulatory layer of GR signaling. GRβ can inhibit GRα-mediated transcription through multiple mechanisms, including competition for glucocorticoid response elements, formation of inactive GRα–GRβ heterodimers, and sequestration of transcriptional coactivators. This dominant-negative effect is most evident for glucocorticoid response element–dependent gene activation and contributes to reduced glucocorticoid responsiveness in inflammatory conditions. In addition, genetic variability within the glucocorticoid receptor gene further contributes to heterogeneity in GR signaling.

Genetic variability in the glucocorticoid receptor gene (NR3C1) adds another layer of influence on GR signaling and systemic responsiveness. Common polymorphisms, including BclI and N363S, are generally associated with increased glucocorticoid sensitivity and have been linked to metabolic phenotypes such as central adiposity, dyslipidemia, hypertension, and increased risk of type 2 diabetes and cardiovascular disease. In contrast, the ER22/23EK variant is associated with relative glucocorticoid resistance and a more favorable metabolic profile. These variants contribute to interindividual differences in metabolic regulation, inflammatory responses, and clinical sensitivity to endogenous and therapeutic glucocorticoids. However, their effects are context-dependent and may vary across disease states and populations, thereby modulating the efficiency of GRα-mediated integration across inter-organ communication systems.

Proinflammatory cytokines (e.g., TNF-α, IL-1) increase GRβ expression and shift the GRα/GRβ ratio, a change associated with glucocorticoid resistance in diseases such as asthma and rheumatoid arthritis. However, GRβ effects are context-dependent and may vary according to cell type, promoter structure, and expression levels, and GRβ also exerts GRα-independent transcriptional regulatory effects [78,79,80].

These findings highlight that the efficacy of GRα-mediated transcriptional control is dynamically modulated by isoform balance and inflammatory signaling environment, providing a mechanistic basis for the development of glucocorticoid resistance during sustained or dysregulated inflammation.

### 5.3. Mechanisms of Glucocorticoid Resistance in Dysregulated Inflammatory Networks

Glucocorticoid receptor (GR) resistance represents a context-dependent impairment of GR signaling observed in chronic inflammation and stress states. This phenomenon arises from converging molecular mechanisms, including persistent activation of proinflammatory transcription factors (NF-κB, AP-1) that interfere with GR-mediated gene regulation, activation of MAPK pathways (p38, JNK) that alter GR phosphorylation and nuclear translocation, and cytokine-driven signaling that disrupts GR–DNA binding and transcriptional activity. Additional contributing factors include post-translational modifications of GR, increased expression of dominant-negative GRβ isoforms, competition for transcriptional coactivators (e.g., p300), and reduced histone deacetylase activity. Together, these mechanisms impair GR’s ability to repress inflammatory gene expression and contribute to reduced glucocorticoid responsiveness in chronic inflammatory diseases [81,82,83].

### 5.4. Induction of Endogenous Anti-Inflammatory Regulators

In addition to transcriptional repression of inflammatory genes, GRα signaling induces expression of anti-inflammatory regulatory proteins that further dampen inflammatory pathways. Among the most important are glucocorticoid-induced leucine zipper (GILZ) and dual-specificity phosphatase-1 (DUSP1/MKP-1). GR-dependent transcription of GILZ inhibits NF-κB and AP-1 signaling through direct interaction with their transcriptional complexes, while DUSP1 attenuates mitogen-activated protein kinase (MAPK) signaling by dephosphorylating p38, JNK, and ERK kinases. Through these complementary mechanisms, GRα integrates transcriptional repression with induction of endogenous inhibitory circuits, thereby sustaining coordinated anti-inflammatory control across tissues [84,85].

### 5.5. Stabilization of Systemic Immune Communication

By modulating inflammatory transcription, immune cell signaling, and cytokine production, GRα stabilizes and fine-tunes the immune–inflammatory communication network, preventing excessive systemic inflammation. In this context, immune signaling functions as an intermediate-speed communication system that links local tissue injury with organism-wide physiological adaptation and coordinated host defense. While GRα-mediated suppression limits pathological inflammation, excessive or prolonged activation may impair host defense, highlighting a context-dependent balance between immune protection and immunosuppression.

## 6. Metabolic Communication System

### 6.1. Metabolic Networks as Inter-Organ Energy Communication

The metabolic communication system coordinates the distribution and utilization of energy substrates across organs during physiological stress. Through integrated signaling among the liver, adipose tissue, skeletal muscle, and endocrine stress pathways, it regulates substrate mobilization and energy allocation across tissues. Within this network, GRα signaling plays a central role in coordinating metabolic responses that sustain systemic energy availability during physiological stress.

### 6.2. Hepatic Gluconeogenesis and Systemic Glucose Mobilization

At the organ level, GRα signaling regulates hepatic gluconeogenesis and systemic glucose mobilization. During fasting or stress, glucocorticoids activate hepatic GR, inducing transcription of key gluconeogenic enzymes—including phosphoenolpyruvate carboxykinase (PEPCK) and glucose-6-phosphatase (G6Pase)—through glucocorticoid response elements (GRE)-dependent mechanisms and interaction with co-regulators such as peroxisome proliferator–activated receptor gamma coactivator 1-α (PGC-1α), forkhead box O1 (FOXO1), and hepatocyte nuclear factor 4-alpha (HNF4α) [20,21,22].

Beyond the liver, GR signaling coordinates whole-body substrate mobilization by promoting skeletal-muscle proteolysis and adipose tissue lipolysis, providing amino acids and glycerol that support hepatic gluconeogenesis. Concurrently, glucocorticoids reduce peripheral glucose uptake, preserving circulating glucose for essential organs such as the brain. Through these coordinated actions, GRα-dependent signaling maintains systemic energy availability and metabolic homeostasis during physiological stress [43,86,87].

Experimental models demonstrate that disruption of hepatic GR signaling markedly impairs gluconeogenic gene activation and reduces hepatic glucose output during fasting, underscoring the essential role of GR in glucose homeostasis [21,88].

### 6.3. Adipose Lipolysis and Fatty Acid Mobilization

Glucocorticoid receptor α (GRα) signaling regulates lipid mobilization during physiological stress by promoting adipose tissue lipolysis and systemic fatty acid release. Glucocorticoids enhance the activity of adipocyte triglyceride lipases, including adipose triglyceride lipase (ATGL) and hormone-sensitive lipase (HSL), and potentiate catecholamine-driven cAMP–protein kinase A (PKA) signaling pathways that stimulate triglyceride hydrolysis [89,90,91]. In parallel, they attenuate insulin’s antilipolytic effects, sustaining the release of non-esterified fatty acids (NEFAs) during stress [92,93].

Through these mechanisms, GR-dependent signaling drives the mobilization of fatty acids and glycerol, providing substrates for hepatic gluconeogenesis and oxidative metabolism in peripheral tissues. In this context, adipose tissue functions as a key metabolic signaling node that connects the liver, skeletal muscle, and endocrine stress pathways to sustain systemic energy availability. Within this integrated network, GRα coordinates systemic energy allocation by integrating endocrine stress signals with metabolic communication across these tissues.

Within the temporal hierarchy of inter-organ communication (Table 3), adipose-derived metabolic signaling operates over minutes to hours, aligning with the Priming and Modulatory phases of homeostatic correction.

## 7. Microbiome Communication System

### 7.1. Microbiome as an Inter-Organ Signaling Network

The microbiome communication system represents an additional layer of inter-organ signaling in which microbial metabolites and microbiota-derived molecular signals modulate host metabolism, immune responses, and epithelial barrier function across multiple organs. Through the production of short-chain fatty acids, bile acid derivatives, microbial peptides, and other metabolites, the intestinal microbiota enables bidirectional communication among the gut, liver, immune system, and central nervous system.

### 7.2. GRα Regulation of Intestinal Barrier Integrity During Inflammatory Stress

Glucocorticoids play an important role in preserving intestinal epithelial barrier integrity during inflammatory stress through GRα–mediated regulation of tight junction proteins and epithelial signaling pathways [62]. Experimental studies in intestinal epithelial models show that glucocorticoids can prevent cytokine-induced barrier disruption by suppressing myosin light chain kinase (MLCK) activation and stabilizing tight junction complexes. In Caco-2 monolayers exposed to TNF-α, glucocorticoids inhibit MLCK-mediated cytoskeletal contraction and prevent increases in paracellular permeability [23].

Additional studies show that glucocorticoids increase transepithelial electrical resistance and promote the expression of sealing tight-junction proteins, such as claudin-4, while suppressing pore-forming claudin-2, thereby enhancing epithelial barrier integrity during inflammatory signaling [24,94]. Similar effects are observed in intestinal organoids from patients with Crohn’s disease, where prednisolone partially restores epithelial structure, junctional organization, and protein expression under inflammatory conditions [95].

Animal models further demonstrate that epithelial GR signaling plays a protective role during intestinal inflammation. Mice lacking epithelial GR develop more severe colitis, increased permeability, and dysregulated expression of barrier-associated genes, underscoring its role in maintaining mucosal barrier homeostasis under inflammatory stress [25].

Although glucocorticoids may weaken barrier function under non-inflamed conditions or chronic stress, most evidence indicates that during inflammation, they promote barrier stabilization, reduce bacterial translocation, and support mucosal homeostasis [96].

Within the inter-organ communication framework, preservation of intestinal barrier integrity represents a key mechanism through which GRα signaling maintains host–microbiome equilibrium and limits systemic inflammatory propagation.

### 7.3. Microbiome-Derived Metabolites and Host Endocrine–Metabolic Signaling

Microbiome-derived metabolites contribute to inter-organ communication by influencing host metabolic, immune, and endocrine signaling pathways, primarily through indirect, host-mediated mechanisms rather than direct receptor-level interactions with GRα. Rather than acting as direct ligands for the glucocorticoid receptor (GR), most metabolites modulate GRα signaling indirectly by altering local glucocorticoid availability, immune tone, and host metabolic regulation.

One key mechanism involves microbiota-dependent regulation of local glucocorticoid production within intestinal tissues. Experimental studies show that germ-free mice exhibit reduced expression of steroidogenic enzymes and diminished intestinal corticosterone production following immune stimulation, despite preserved adrenal responses, indicating that the microbiota regulates local glucocorticoid availability and tissue-specific GRα activation [97]. Conversely, glucocorticoid exposure reshapes gut microbial composition and alters circadian glucocorticoid rhythms, highlighting a bidirectional interaction between microbial communities and host glucocorticoid signaling [98,99,100]. These effects are bidirectional and mediated through host regulatory pathways, rather than reflecting direct GRα–microbiome signaling interactions.

Microbial metabolites also interact with host signaling pathways that functionally intersect with GRα-regulated processes. Short-chain fatty acids (SCFAs), including acetate and propionate, activate metabolite-sensing G protein–coupled receptors such as GPR41 and GPR43 on epithelial, immune, and enteroendocrine cells, modulating inflammatory signaling and systemic metabolic regulation [44,101].These pathways functionally converge with glucocorticoid-regulated metabolic and inflammatory programs, including NF-κB–dependent immune responses and enteroendocrine hormone secretion. Alterations in SCFAs during glucocorticoid exposure have been associated with impaired glucagon-like peptide-1 (GLP-1)secretion and metabolic dysregulation, highlighting functional overlap between microbial metabolite signaling and glucocorticoid-regulated metabolic pathways [102].

Additional microbial metabolites—including indole derivatives, bile acids, and polyamines—interact with host nuclear and G protein–coupled receptors such as the aryl hydrocarbon receptor (AhR) and the farnesoid X receptor (FXR). These pathways regulate epithelial barrier integrity, immune responses, and metabolic homeostasis, thereby shaping the physiological context in which GRα signaling operates [45,103,104].

Through these mechanisms, microbiome-derived metabolites influence systemic physiology by influencing inflammatory signaling, metabolic regulation, and epithelial barrier function. Within the inter-organ communication framework, the microbiome functions as a dynamic signaling interface that links intestinal microbial activity with host endocrine, immune, and metabolic stress responses.

### 7.4. Integration of Microbiome and Metabolic Communication

Because metabolic substrates and microbial metabolites are closely interconnected within the intestinal environment, inter-organ metabolic communication is functionally coupled to microbiome-derived signaling pathways that regulate host metabolism, immune responses, and epithelial barrier function.

Within this integrated network, GRα signaling maintains host–microbiome homeostasis by coordinating epithelial barrier integrity, immune regulation, and metabolic adaptation across the gut–liver–immune axis.

## 8. Mechanical Communication System 

### 8.1. Mechanical Forces as Biological Communication Signals

The mechanical communication system represents a fundamental mechanism through which physical forces generated within tissues transmit biological information across cells and organs. Mechanical signals arise from diverse physiological processes, including blood flow–induced shear stress, tissue stretch during respiration and cardiac contraction, extracellular matrix tension, and changes in tissue stiffness during inflammation or repair. These forces are detected by mechanotransduction pathways that convert physical stimuli into biochemical signals regulating gene expression, cellular behavior, and tissue remodeling.

### 8.2. Cellular Mechanotransduction Pathways

Mechanotransduction occurs through multiple molecular sensors, including integrins, cytoskeletal structures, mechanosensitive ion channels, and nuclear–cytoskeletal linkages that connect the extracellular matrix to chromatin. Through these pathways, physical forces regulate key cellular processes—including proliferation, differentiation, migration, and extracellular matrix production, thereby influencing tissue architecture and organ function.

### 8.3. GRα Modulation of Mechanotransduction Signaling

GRα signaling modulates mechanosensitive pathways in multiple cell types, including fibroblasts, epithelial cells, endothelial cells, and lung mesenchymal cells. Experimental studies show that glucocorticoids suppress mechanically induced gene expression programs through transcriptional and post-transcriptional regulation of mechanosensitive signaling pathways. For example, glucocorticoid signaling counteracts cellular responses to mechanical stretch through GR-dependent mRNA decay of mechanosensitive regulators such as EGR1, CITED2, and BMP7, thereby limiting stretch-induced proliferation and matrix remodeling [26].

In epithelial and airway cells, glucocorticoids can also influence mechanosensitive signaling through rapid non-genomic pathways. Membrane-associated GRs activate PLC/PKC and GSK-3β signaling cascades that regulate β-catenin–dependent pathways controlling epithelial migration and wound repair [27]. In models of airway mechanical stress, dexamethasone attenuates stretch-induced activation of mechanosensitive ion channels such as transient receptor potential canonical-1 (TRPC1) and reduces downstream inflammatory and remodeling responses [28].

### 8.4. Mechanical Regulation of Vascular and Developmental Remodeling

Mechanical signaling pathways are also closely linked to vascular and developmental processes. In endothelial cells, GR signaling restrains canonical Wnt/β-catenin activity, thereby modulating angiogenesis, endothelial-to-mesenchymal transition, and vascular remodeling—processes that influence extracellular matrix organization, tissue stiffness, and biomechanical signaling within the microenvironment [105,106]. During lung development, mesenchymal GR signaling regulates pathways such as WNT, VEGF, and JAK–STAT that control extracellular matrix organization and alveolar maturation, thereby shaping the mechanical properties required for effective respiratory function [107].

### 8.5. Mechanical Signaling in Tissue Repair and Structural Adaptation

Through these mechanisms, glucocorticoid signaling interacts with mechanotransduction networks to regulate tissue architecture, vascular remodeling, and epithelial repair. Within the inter-organ communication framework, mechanical signaling represents a relatively slower but essential communication pathway through which physical forces coordinate structural adaptation, tissue regeneration, and restoration of normal organ function.

### 8.6. GRα Integration of Mechanotransduction and Tissue Remodeling 

Glucocorticoid receptor α (GRα) signaling modulates mechanotransduction pathways by integrating mechanical and inflammatory cues that regulate tissue remodeling during physiological stress and recovery. Experimental evidence indicates that glucocorticoids influence cytoskeletal organization, extracellular matrix turnover, and fibroblast behavior through transcriptional and non-genomic mechanisms involving NF-κB and MAPK signaling pathways. GRα can repress NF-κB– and MAPK-dependent inflammatory signaling through transrepression and induction of MAPK phosphatases, while also engaging in context-dependent crosstalk that shapes cellular responses to combined mechanical and inflammatory stimuli [81,108,109].

GRα signaling also influences fibroblast function and extracellular matrix organization, regulating adhesion, migration, and matrix remodeling processes that determine tissue architecture. Changes in GR signaling alter fibroblast morphology and extracellular matrix composition, thereby modifying the biomechanical properties of the tissue microenvironment.

Through interactions with cytoskeletal elements, including microtubule-associated transport mechanisms, GRα signaling is functionally linked to cellular structural dynamics underlying mechanotransduction [110].

In this context, GRα acts as a systems-level integrator linking mechanical communication with inflammatory, metabolic, endocrine, and vascular signaling networks, particularly during the Restorative phase of homeostatic correction.

Within the temporal hierarchy of inter-organ communication (Table 3), mechanical signaling operates over hours to weeks and contributes predominantly to the Restorative phase of homeostatic correction, supporting tissue remodeling and recovery of organ structure and function. Given the integrative and conceptual nature of this framework, several limitations should be acknowledged.

## 9. Limitations

This manuscript presents a hypothesis-driven, integrative framework rather than a formal systematic review or meta-analysis. Its purpose is to synthesize experimental, translational, and clinical evidence into a systems-level model of inter-organ communication. As such, the strength of supporting evidence varies across communication systems, with some domains supported primarily by mechanistic and preclinical studies.

Evidence acquisition incorporated AI-assisted literature searches using the Consensus platform. While this enabled structured, hypothesis-driven identification of relevant studies, it introduces potential bias related to algorithmic ranking, incomplete retrieval, and limited reproducibility. These limitations were mitigated through manual verification using primary biomedical literature (PubMed), although some degree of selection bias cannot be excluded.

Finally, the characterization of GRα as a systems-level integrator reflects the synthesis of converging evidence rather than uniform direct mechanistic validation across all communication domains. Accordingly, the proposed integrative role of GRα should be interpreted as a systems-level conceptual model supported by heterogeneous lines of evidence, rather than a fully delineated hierarchical regulatory framework. This framework is intended to guide future investigation rather than provide a definitive mechanistic description of all inter-organ interactions.

An additional limitation is that this framework does not explicitly address sex differences in GR signaling. Substantial evidence indicates that glucocorticoid signaling and HPA axis regulation differ between sexes, influencing immune, metabolic, vascular, and behavioral responses. These differences may modulate GRα-mediated inter-organ communication and systemic regulation within this conceptual framework. Future work should incorporate sex-specific analyses to further refine and validate this model.

## 10. Summary and Conclusions

Taken together, the findings in this review support a network-based model of systemic physiology in which GRα functions as a systems-level integrator of signals across multiple inter-organ communication systems, coordinating adaptive responses that maintain and restore organismal homeostasis. In this model, physiological regulation arises from coordinated signaling across interconnected networks rather than independent organ activity. Neural, endocrine, immune, vascular, lymphatic, metabolic, microbiome–gut, and mechanical systems continuously exchange biological information, enabling coordinated organism-wide responses that regulate host defense, energy allocation, tissue repair, and adaptation to stress across basal physiological conditions and during physiological challenge.

Within this integrated architecture, GRα serves as a central molecular integrator of inter-organ communication networks. Converging evidence supports its role as a systems-level regulator, although the strength of evidence varies across domains and includes mechanistic, translational, and clinical observations. Because it is expressed in nearly all nucleated cells, GRα coordinates transcriptional programs that regulate inflammation, maintain vascular integrity, mobilize metabolic substrates, support epithelial barrier function, preserve organ-specific functional integrity both under baseline conditions and during physiological stress, and drive tissue remodeling. Through these actions, GRα links endocrine stress signaling with neural, immune, metabolic, and structural communication systems to sustain systemic homeostasis.

Critical illness represents a state in which this integrated network becomes profoundly disrupted. Dysregulated signaling across vascular, immune, metabolic, and epithelial pathways leads to loss of coordinated regulation, impaired homeostasis, and progressive multi-organ dysfunction. Understanding these interactions provides a framework for identifying key molecular regulators, such as GRα, that may contribute to restoring coordinated physiological responses during severe systemic stress.

This systems-level model of physiology emphasizes that maintenance and restoration of organismal homeostasis depend on continuous integration of signals across multiple inter-organ communication networks. Within this framework, GRα emerges as a key regulatory node that coordinates transcriptional responses across distributed physiological systems, supporting the stabilization and recovery of systemic physiology during health, physiological stress, and critical illness.

## Figures and Tables

**Table 1:** Structured AI-Assisted Query Framework for Hypothesis-Driven Evidence Acquisition Across Inter-Organ Communication Systems.

Communication System	Evidence Domain	Representative Consensus Query	Purpose of Query
Vascular	Endothelial barrier regulation	Does endothelial glucocorticoid receptor preserve vascular barrier integrity during inflammation?	Identify evidence for GR signaling regulation of endothelial barrier stability
Vascular	Vascular tone regulation	How do glucocorticoids regulate endothelial nitric oxide signaling and vasoregulatory pathways (e.g., eNOS)?	Evaluate GR signaling control of endothelial vasoregulatory pathways
Vascular	Leukocyte trafficking	Do glucocorticoids suppress endothelial NF-κB activation and adhesion molecules?	Assess GR signaling effects on inflammatory endothelial activation
Vascular	Cardiovascular responsiveness	Do glucocorticoids enhance cardiovascular responsiveness by regulating adrenergic receptor expression or myocardial contractility?	Assess GR signaling influence on cardiac output and vascular tone
Neural	Autonomic regulation	What role does the glucocorticoid receptor play in autonomic nervous system regulation?	Identify GR signaling regulation of autonomic and neural stress circuits
Neural	Stress responses	Does GR regulate catecholamine synthesis or sympathoadrenal stress responses?	Evaluate GR signaling influence on adrenal catecholamine production
Lymphatic	Lymphangiogenesis	Do glucocorticoids regulate lymphatic vessel growth or lymphangiogenic signaling pathways (e.g., VEGF-C/VEGFR-3)?	Assess GR signaling influence on lymphatic vessel development and signaling
Lymphatic	Immune trafficking	Does GR signaling regulate immune cell trafficking through lymphatic vessels and lymph nodes?	Identify GR signaling effects on lymphatic immune transport
Lymphatic	Lymphatic pumping/transport	Do glucocorticoids regulate lymphatic vessel contractility or lymphatic pumping that controls lymph flow?	Assess GR signaling influence on lymphatic transport dynamics
Endocrine	Circadian coordination	How do glucocorticoids entrain peripheral circadian clocks?	Evaluate GR signaling regulation of peripheral circadian oscillators
Endocrine	Systemic hormonal coordination	How does GR signaling coordinate systemic endocrine stress responses across organs?	Assess GR signaling integration of endocrine stress signaling
Endocrine	Stress adaptation	Do glucocorticoids synchronize peripheral metabolic and immune rhythms through circadian GR signaling?	Identify links between GR signaling and systemic circadian adaptation
Endocrine	Genetic variability/receptor sensitivity	How do NR3C1 polymorphisms influence GR sensitivity and systemic metabolic or inflammatory responses?	Assess how genetic variation modifies GR signaling and interindividual response to endocrine stress signals
Immune	Inflammatory transcription	Does GR signaling suppress NF-κB or AP-1 inflammatory transcription across immune cells?	Evaluate GR repression of pro-inflammatory transcription factors
Immune	Resolution pathways	Does GR induce anti-inflammatory mediators such as GILZ or DUSP1/MKP-1?	Assess GR signaling activation of inflammation-resolving pathways
Immune	Cytokine network integration	Does GR signaling coordinate systemic cytokine networks across multiple organs during inflammation?	Assess GR signaling role in inter-organ inflammatory communication
Immune	Transcriptional crosstalk	How does GR interact with NF-κB and AP-1 to regulate inflammatory gene expression in different tissues?	Evaluate GR-mediated transcriptional integration across immune pathways
Metabolic	Energy allocation	Does GR signaling regulate hepatic gluconeogenesis and systemic glucose mobilization during physiological stress?	Identify GR signaling control of stress-induced glucose mobilization
Metabolic	Lipid metabolism	How do glucocorticoids regulate lipolysis and fatty acid mobilization during systemic stress responses?	Evaluate GR signaling control of lipid mobilization
Metabolic	Multi-organ energy coordination	Does GR signaling coordinate energy substrate distribution across liver, muscle, and adipose tissue during stress?	Assess GR signaling role in systemic metabolic integration
Metabolic	Hormonal interaction	How do glucocorticoids interact with insulin, glucagon, and catecholamines to regulate systemic metabolism?	Evaluate GR signaling coordination of endocrine–metabolic signaling
Microbiome–gut	Barrier integrity	Do glucocorticoids preserve intestinal epithelial barrier integrity during inflammatory stress?	Assess GR signaling effects on epithelial barrier stability
Microbiome–gut	Host–microbiome signaling	How do microbiome-derived metabolites interact with GR signaling pathways?	Identify bidirectional signaling between microbiome metabolites and GR pathways
Mechanical–structural	Mechanotransduction	Does GR signaling regulate mechanotransduction pathways in endothelial, epithelial, or lung cells?	Evaluate GR signaling influence on cellular mechanosensing pathways
Mechanical–structural	Tissue remodeling	How do glucocorticoids influence extracellular matrix remodeling and fibroblast activation during tissue repair?	Assess GR signaling regulation of structural repair and fibrosis pathways
Mechanical–structural	Cytoskeletal regulation	Does GR signaling regulate cytoskeletal organization and mechanosensitive pathways in fibroblasts and epithelial cells?	Assess GR signaling effects on cellular mechanotransduction
Mechanical–structural	ECM biomechanics	How do glucocorticoids influence extracellular matrix stiffness and tissue biomechanics during repair and inflammation?	Evaluate GR signaling regulation of tissue mechanical properties
Extracellular vesicle	Vesicle-mediated signaling	Do glucocorticoids or GR signaling regulate extracellular vesicle or exosome release and cargo involved in intercellular communication?	Assess GR signaling influence on vesicle-mediated intercellular signaling
Cross-system integration	Systems biology	Does GR signaling act as a systems-level integrator of inter-organ communication networks?	Assess evidence supporting GR signaling as a system-level integrator of organism-wide responses
Cross-system/Receptor regulation	GR isoform modulation	Does glucocorticoid receptor beta (GRβ) inhibit GRα-mediated transcription and contribute to glucocorticoid resistance?	Assess role of GR isoform balance in regulating glucocorticoid signaling and systemic responsiveness
Cross-system/Receptor regulation	Chaperone-mediated GR regulation	What is the role of chaperone complexes (HSP90, HSP70, FKBP51, FKBP52) in glucocorticoid receptor folding and signaling?	Assess regulation of GR signaling activation, intracellular trafficking, and signaling efficiency
Cross-system/Receptor regulation	Glucocorticoid resistance mechanisms	What mechanisms cause GR resistance in chronic inflammation or stress (e.g., NF-κB, AP-1 interference)?	Assess molecular pathways impairing GR signaling and reducing glucocorticoid responsiveness

Legend: This table presents the structured, hypothesis-driven queries used to guide AI-assisted literature searches using the Consensus research platform. Queries were organized by inter-organ communication systems and designed to evaluate whether glucocorticoid receptor α (GRα) signaling regulates key functional domains within each system. This approach establishes a systematic and reproducible strategy for evidence acquisition, enabling targeted evaluation of GRα-mediated regulation across vascular, neural, endocrine, immune-inflammatory, lymphatic, metabolic, microbiome–gut, and mechanical communication pathways. Additional queries were incorporated iteratively to capture emerging concepts, including the role of GRα signaling as a systems-level integrator of inter-organ communication networks.

**Table 2:** Major Inter-Organ Communication Systems Coordinating Systemic Homeostasis and Their Integration by Glucocorticoid Receptor α (GRα).

Communication System	Primary Signals	Representative Physiological Functions	GRα Regulatory Roles	Key References
Neural	Electrical impulses, neurotransmitters	Rapid systemic coordination of stress responses; autonomic regulation; behavioral adaptation	GRα mediates HPA axis feedback, regulates autonomic stress circuits, entrains circadian signaling	[5,6,7]
Endocrine	Hormones (glucocorticoids, ACTH, etc.)	Systemic hormonal coordination, circadian entrainment, metabolic regulation	GRα transduces endocrine glucocorticoid signals across organs and synchronizes peripheral clocks	[8,9,10]
Vascular	Endothelial mediators, circulating cells	Inter-organ transport of oxygen, nutrients, immune mediators	GRα stabilizes endothelial barrier integrity, regulates NO signaling, suppresses inflammatory activation	[11,12,13]
Lymphatic	Immune cell trafficking, antigen transport	Tissue–immune communication, lymphatic drainage, inflammatory regulation	GRα regulates lymphangiogenic signaling through suppression of VEGF-C	[14,15,16]
Immune	Cytokines, chemokines, alarmins	Host defense, systemic inflammatory coordination	GRα represses NF-κB/AP-1 signaling; induces GILZ and DUSP1	[17,18,19]
Metabolic	Glucose, fatty acids, amino acids	Energy mobilization and allocation during stress	GRα regulates hepatic gluconeogenesis and systemic substrate mobilization	[20,21,22]
Microbiome	SCFAs, bile acids, microbial metabolites	Gut–immune–metabolic communication	GRα maintains epithelial barrier integrity and modulates microbiome–host signaling	[23,24,25]
Mechanical	Shear stress, stretch, ECM tension	Mechanotransduction regulating tissue remodeling	GRα modulates mechanosensitive transcription and ECM remodeling	[26,27,28]

Legend: This table defines the major inter-organ communication systems that coordinate systemic homeostasis and illustrates how glucocorticoid receptor α (GRα) is proposed to function as a central molecular integrator across these networks, based on converging mechanistic and physiological evidence. Each system transmits distinct classes of signals—electrical, hormonal, vascular, immune, metabolic, microbial, or mechanical—that regulate inter-organ information flow. GRα is proposed to integrate these signals through context-dependent transcriptional and non-genomic mechanisms, including repression of NF-κB/AP-1–mediated inflammation, regulation of metabolic gene programs, stabilization of endothelial and epithelial barriers, modulation of neuroendocrine feedback loops, and control of mechanotransduction and extracellular matrix remodeling. Through these coordinated actions, GRα is proposed to synchronize inter-organ communication, thereby contributing to the maintenance of energy availability, the regulation of immune responses, and the preservation of tissue integrity during physiological stress and recovery.

**Table 3:** Temporal hierarchy and phase-specific organization of inter-organ communication systems.

Communication System	Primary Signals	Typical Response Time	Physiological Role	Primary Functional Phase (Homeostatic Correction)
**Neural**	Electrical impulses, neurotransmitters	milliseconds–seconds	Immediate detection of threat and reflex coordination [5,6]	Priming
**Vascular (circulatory)**	Blood flow dynamics, circulating mediators, endothelial signals	seconds–minutes	Rapid systemic distribution of oxygen, hormones, and immune mediators [12,13]	Priming → Modulatory
**Immune–inflammatory**	Cytokines, chemokines, DAMPs/PAMPs	minutes–hours (can extend to days)	Detection of injury and orchestration of host defense [17,19]	Priming → Modulatory
**Endocrine–hormonal**	Hormones, circadian signals	seconds–hours	System-wide regulation of metabolism and stress adaptation [8,9]	Priming → Modulatory → Restorative
**Metabolic**	Metabolic intermediates, substrates	minutes–hours (dynamic, context-dependent)	Allocation of energy resources across organs [21,22]	Priming → Modulatory → Restorative
**Lymphatic**	Immune cells, antigens, interstitial fluid	hours–days	Immune surveillance and fluid homeostasis [15,16]	Modulatory → Restorative
**Microbiome–gut**	Microbial metabolites, mucosal signals	hours–days	Modulation of systemic immunity and metabolism [23,25]	Modulatory → Restorative
**Mechanical–structural**	Shear stress, ECM remodeling signals	hours–weeks	Tissue remodeling and structural repair [26,27]	Restorative

Legend: This table illustrates the temporal hierarchy and phase-specific organization of inter-organ communication systems through which biological signals are transmitted and integrated across the organism. Rapid neural and vascular responses initiate systemic coordination during the Priming Phase, followed by endocrine, immune, and metabolic pathways that regulate intermediate adaptations during the Modulatory Phase, and culminating in lymphatic, microbiome, and mechanical systems that support tissue repair and restoration during the Restorative Phase. The vascular system functions as a central distribution network linking rapid neural activation to endocrine, immune, and metabolic signaling across organs, facilitating the spatial propagation and amplification of systemic responses.

**Table 4:** Cross-System Coupling of Inter-Organ Communication Networks During Acute Infection.

Communication System	Primary Signals/Mediators	Role During Acute Infection	Major Cross-System Interactions
Neural	Autonomic signaling, vagal pathways, catecholamines	Rapid detection of inflammatory signals; autonomic coordination of fever, sickness behavior, and stress responses.	Activates endocrine (HPA axis), modulates immune responses via inflammatory reflex, regulates vascular tone [5,40]
Vascular	Endothelial mediators (NO, prostacyclin), adhesion molecules	Endothelial activation, leukocyte trafficking, microcirculatory redistribution, vascular permeability.	Facilitates immune cell migration, interacts with lymphatic drainage, influences mechanical stress through edema [12,41]
Lymphatic	Lymph flow, antigen transport, chemokines	Transport of antigen and immune cells to lymph nodes for activation of adaptive immunity.	Coordinates with immune activation, maintains fluid balance with vascular system, communicates with mucosal immunity [15,16]
Endocrine–Hormonal	CRH, ACTH, cortisol, catecholamines	HPA axis activation and systemic hormonal coordination of stress responses and GRα activation.	Regulates immune inflammation, mobilizes metabolic substrates, stabilizes vascular signaling [8,42]
Immune–Inflammatory	Cytokines (TNF, IL-1, IL-6), TLR signaling, NF-κB	Pathogen detection, cytokine release, leukocyte activation, initiation of inflammatory responses.	Activates neural sensing, triggers endocrine stress response, drives vascular and metabolic adaptations [17,19]
Metabolic	Glucose, lactate, ketones, lipid mobilization	Energy mobilization supporting immune activation and tissue defense.	Interacts with endocrine hormones, fuels immune responses, supports mechanical tissue repair [21,43]
Microbiome–Gut	Microbial metabolites (SCFAs), barrier signaling	Modulation of immune tone and maintenance of epithelial barrier integrity.	Influences immune signaling, interacts with metabolic regulation and neural gut–brain pathways [44,45]
Mechanical	Tissue stretch, extracellular matrix stress, mechanotransduction	Mechanical stress responses associated with inflammation, edema, and tissue remodeling.	Feeds back to immune activation, alters vascular responses, supports tissue repair processes [26,46]

Legend: This table summarizes how the major inter-organ communication systems interact during acute infection. Signals generated within one physiological network rapidly propagate across neural, endocrine, immune, vascular, metabolic, lymphatic, microbiome, and mechanical pathways, producing coordinated organism-wide responses that regulate host defense, energy mobilization, vascular stability, and tissue repair.

## Data Availability

No new data were created or analyzed in this study.
